# Effect of fermented milk *Pediococcus acidilactici* BK01 on cholesterol and microbiota in Wistar mice intestine

**DOI:** 10.5455/javar.2023.j653

**Published:** 2023-03-31

**Authors:** Sri Melia, Indri Juliyarsi, Yulianti Fitri Kurnia, Salam N. Aritonang, Endang Purwati, Ade Sukma, Najmiatul Fitria, Susmiati Susmiati, Malinda Meinapuri, Yudha Endra Pratama, Nurazizah Ramadhanti

**Affiliations:** 1 Faculty of Animal Science, Universitas Andalas, Padang, Indonesia; 2 Department of Pharmacology and Clinical Pharmacy, Faculty of Pharmacy, Universitas Andalas, Padang, Indonesia; 3 Faculty of Nursing, Universitas Andalas, Padang, Indonesia; 4 Faculty of Medicine, Universitas Andalas, Padang, Indonesia; 5 Magister Program of Animal Science, Universitas Andalas, Padang, Indonesia

**Keywords:** Fermented milk, *Pediococcus acidilactici* BK01, probiotic, cholesterol

## Abstract

**Objective:**

This study examines the effect of fermented milk containing the probiotic *Pediococcus acidilactici* BK01 on cholesterol and intestinal microbiota.

**Materials and Methods:**

24 male rats weighing an average of 200 gm each spent 1 week in a cage adapting to their new environment. They were fed standard feed daily and were allowed to drink *ad libitum*. For 3 weeks, rats were divided into four groups (doses of fermented milk): M+ (control), M1 (0.35 ml), M2 (0.70 ml), and M3 (1.05 ml). The analysis includes bodyweight determination, serum biochemical analysis, and intestine microbiota analysis.

**Results:**

The results indicated that while *P. acidilactici* BK01 fermented milk did not affect body weight or high-density lipoprotein, it did have a beneficial effect on total serum cholesterol and triglyceride levels. Additionally, treatment of fermented milk with *P. acidilactici* BK01 has been shown to increase the total lactic acid bacteria (LAB) in the intestine, as indicated by changes in the intestinal villi.

**Conclusion:**

Administering fermented milk (*P. acidilactici* BK01, 1.05 ml) can reduce total serum cholesterol and increase the number of LAB in intestinal villi in experimental animals, so it has the potential to be a probiotic.

## Introduction

Fermented milk containing lactic acid bacteria (LAB) is widely consumed due to its probiotic benefits for health. The World Health Organization defines probiotics as live bacteria that promote the host’s health [1]. LAB found in fermented foods has been shown to have therapeutic effects on human health, with improvements in hepatic illness, allergies, hypertension, cancer, blood cholesterol, and hyperlipidemia [2]. Hypercholesterolemia, known as high cholesterol, is one of the main factors in atherosclerosis and other cardiovascular diseases. Probiotic applications for cholesterol-controlling purposes have become popular in the past because of their contributions to human health [3]. Several *Lactobacillales*,* Bifidobacteriales*, yeasts, and *Bacillus* species have been widely known, recognized, and utilized as probiotics. Potential benefits of probiotics involve but aren’t limited to, gastrointestinal microbe stability, pathogen inhibition, immunotherapeutic activity, hypocholesterolemic action, relief from constipation, allergic rhinitis, lactose intolerance, irritable bowel syndrome, and colon cancer [4].

Many types of LAB were isolated from various local foods in Indonesia. For example, dadih, a typical food from West Sumatra, contains *Lactobacillus plantarum *[5], *Lactococcus lactis *ssp. *lactis*, *Lactococcus lactis *ssp. *cremoris*, *Lactiplantibacillus pentosus, *and *Pediococcus pentosaceus *[6]*,* *Lactobacillus casei *subsp. *casei *R-68 (LCR-68) [7], *Lactobacillus brevis, L. lactis *subsp.* lactis*,* Leuconostoc mesenteroides*,* L. casei*,* L. plantarum *subsp.* plantarum*,* Limosilactobacillus fermentum*,* E. faecium*, and *Lacticaseibacillus rhamnosus *[8]. *Lactiplantibacillus pentosus *HBUAS53657 is a probiotic isolated from buffalo milk [9].

In addition, Bekasam, a fermented fish product from South Sumatra, contains LAB such as *L. plantarum *T2565*, L. plantarum *B765*, L. plantarum *B1465*, L. plantarum *N2352, *P. pentosaceus *B1666, *L. pentosus *B2555 [10], and *Pediococcus acidilactici *BK01 [11]. *Lactobacillus fermentum* strain CAU6337 is a lactic acid bacterium found in tempoyak, a fermented food traditionally prepared from durian (*Durio zibethinus*) pulp [12]. *Lactobacillus fermentum* KF7 was found in virgin coconut oil [13].

The previous research revealed that LAB is essential in reducing cholesterol for human health. Moon et al. [14] showed the isolation of *P. acidilactici *M76 *makgeolli*. *Makgeolli *has an alcohol content of 6%–10%. In addition, these microorganisms were administered to animals fed a high-fat meal. In mice fed a high-fat diet, *P. acidilactici *M76 decreased lipid accumulation*. *Furthermore, several kinds of research state that numerous species *of L. plantarum* have the same ability to reduce hyper-cholesterol [15–18]. Moreover, other LAB, like *L. fermentum *noted a capacity to decrease high cholesterol [19–21] *in-vivo* in male mice [22] and 8-week-old hamsters [3].

Yadav et al. [19] revealed that *L. fermentum* MTCC 5898 has probiotic potential, which is consumed to resolve hyper-cholesterol. Furthermore, several researchers confirmed that various *L. plantarum *was the probiotic candidate with decreasing cholesterol activity via bile acid deconjugation modulatory [15,16]. Moreover, high-cholesterol diet mice supported by *L. plantarum *TAR4 significantly reduced total serum cholesterol. Recently, Daliri et al. [23] explored *P. acidilactici* SDL 1402, *P. acidilactici *SDL 1405, *Weissella cibaria *SCCB2306, and *L. rhamnosus *JDFM6 isolated from Korean soya beans as a probiotic candidate to prevent hypercholesterolemia. This research objective was to investigate *in vivo* the influence of fermented milk (*P. acidilactici* BK01) isolated from fermented fish (Bekasam) on Wistar mice via cholesterol and microbiota.

## Materials and Methods

### Ethical approval

All experimental protocols were approved by the Health Research Ethics Committee of Andalas University Padang (No. 009/laiketik/KEPKFKEPUNAND).

### Animal maintenance

Through 1 week, 24 male Wistar mice weighed approximately 200 gm on average and were acclimated to their environment in cages with access to standard food and water. Mice were cared for in a room with a temperature of 20°C–22°C, a humidity of 60%–70%, dark lighting at night, and sunlight during the day. Mice were kept in cages and then divided into four groups (doses of fermented milk): Group M+ (control), Group M1 (0.35 ml), Group M2 (0.70 ml), and Group M3 (1.05 ml). All the mice were treated with normal feed-supported fat from egg yolks for 3 weeks. 

### Fermented milk preparation

Pasteurized goat milk was heated to 65°C–67°C for 30 min before reaching 37°C. The *P. acidilactici* BK01 (1.27 × 10^9^ CFU/ml). The starter culture was added at a concentration of up to 5%, and it was incubated at 37°C for 12 h. The fermented milk of *P. acidilactici* BK01 was kept [24].

### Serum biochemical analysis

Samples of blood were taken from the orbital venous plexus of the mice after anesthesia was administered. Blood serum was separated using a 4,000-rpm centrifuge at 4°C for 20 min (EBA-20) and stored at −20°C until further analyses. Cholesterol analyses were determined using commercial assay kits (Greiner Diagnostic GmbH, Bahlingen, Germany) [25].

### Microflora analysis in the intestine

Small intestines were taken from mice dissected after 4 weeks of a fermented milk diet and 0.85% NaCl. Prepare a 1 ml sample and dissolve it in 9 ml of de Man Rogosa Sharpe (MRS) Broth (Merck TM, Germany) in a test tube. Use buffered peptone water (Merck^TM^, Germany) and vortex until homogenous for aerobic microorganisms. Then, successive dilutions were carried out. The samples were planted in a Petri dish containing MRS Agar (Merck^TM^, Germany) using the spread method for lactic acid before being flattened with an alcohol-soaked and burned hockey stick. Then, using an anaerobic container, make the inoculum, incubate at 37°C for 48 h, and label each Petri dish. After 48 h, the Quebec Colony Counter measured the expanding colony.

### Small intestine preparations

The tissue was placed on a tissue cassette, then soaked in 70% alcohol for one night. After that, the ileal tissue was dehydrated with graded alcohol and purified in paraffin. Paraffin blocks were cut 4–4 µm wide and pasted on slides. Then deparaffinized with xylol solution for 3 min and dehydrated with alcohol. After that, it was processed with hematoxylin-eosin staining [26]. Histopathological changes were observed using a light microscope with 100× dilatation.

### Statistical analysis

Statistical Package for the Social Sciences was used to analyze the data, and Duncan’s multiple range test found statistically significant differences in the group. A significance level of *p*-value 0.05 was used.

## Results and Discussion

### Weight

The administration of *P. acidilactici* BK01 fermented milk to mice at doses up to 1.05 ml (M3) had no significant (p > 0.05) effect on the body weight of rats after 3 weeks as compared to the Control (M+) group (Table 1). The fermented milk in this study contained about 85% water, 3.5% protein, and 3.6% fat [24]. The content of nutrients in this fermented milk did not cause weight gain in mice after 3 weeks. Giving fermented milk P. acidilactici BK01 with a dose of 1.05 ml can reduce total serum cholesterol and increase the number of bacteria in intestinal villi in animal models, so it has the potential to be a probiotic drink.

Yang et al. [3] showed a similar pattern and declared that *L. fermentum* ZJU IDS06 and *L. plantarum* ZY08 did not support weight gain. On the contrary, Wang et al. [20] proved *Lactobacillus* was potent for losing weight after 9 weeks. Furthermore, *Lactobacillus* as a probiotic reduces obesity in mice on a high-fat diet through fat accumulation, lipid metabolism, and adjusted leptin and adiponectin content. In addition, Chen et al. [28] explained that *Lactobacillus* decreases weight gain acceleration, transforms body fat, and lessens lever tissue weight and TG level. Recently, [18] stated that *L. plantarum* S9 decreased extra weight gain in mice after week 8, and *L. fermentum* and *L. rhamnosus* prevented weight gain after 30 days [21].

### Total serum cholesterol

Table 2 shows that *P. acidilactici* BK01 fermented milk significantly reduced total serum cholesterol. Total serum cholesterol levels in fermented milk were not different from 1.05 ml (M3) in control (M). After 3 weeks of giving fermented milk, total serum cholesterol decreased by 30%. It’s the same with [17], where the high-cholesterol diet mice supported by *L. plantarum *TAR4 significantly reduced total serum cholesterol (29.55%) [25]. *Pediococcus pentosaceus* strain KID7 (3 × 10^8^ CFU/ml) and [27], *L. plantarum* 9-41-A, and *L. fermentum* M1-16 administered to mice on a high-cholesterol diet can lower total serum cholesterol. Furthermore, Yang et al. [3] stated that supplementing the *L. fermentum *ZJUIDS06 diet decreased total cholesterol in hamsters for 8 weeks.

Molinero et al. [29] explained that the various microbiota in the intestine has metabolism activities that reduce cholesterol, but even the actual mechanism of reducing cholesterol still needs to be explained. However, the microbiota can absorb some cholesterol to prevent intestine captivity. Supporting an enzyme that converts cholesterol to unabsorbable koprosterol, which is then excreted directly, reduces cholesterol reabsorption associated with bile acid, which changes the pH of the intestine to digestive and fat immersion, including cholesterol. Furthermore, Baccouri et al. [30] explained that the peptidoglycan cell wall of probiotic bacteria binds the cholesterol even to dead bacteria to decrease the levels. Moreover, Shehata et al. [31] describe the cholesterol-binding mechanism related to exopolysaccharide activity in the media and the gut. In addition, Palaniyandi et al. [22] enlighted the effect of hypercholesterolemia from *L. fermentum *MJM60397 related to bile acid deconjugation activity that led to decreasing bile acid absorption and increasing bile acid on feces, which makes the *L. fermentum *MJM60397 capable of being developed into a potential anti-hypercholesterol serum.

### High-density lipoprotein (HDL) cholesterol

Table 3 reveals that *P. acidilactici* BK01 fermented milk did not affect HDL cholesterol (*p* > 0.05). The results from this research corroborate those reported previously [32,33]. During administration, there was no change in HDL levels in high-cholesterol-fed mice or *L. plantarum*. Similarly, *L. plantarum* 9-41-A or *L. fermentum* M1-16 didn’t affect HDL in high-cholesterol-fed rats [27]. Jang et al. [34] also stated that the administration of *P. acidilactici* FS2 in mice did not cause changes in HDL. *Lacticaseibacillus rhamnosus* FM9 and *L. fermentum* Y57 reduce HDL [21] L. fermentum ZJUIDS06 did not affect the HDL level of the gold hamster [3].

### Triglycerides (TG)

Table 4 shows that *P. acidilactici* BK01 fermented milk reduced TG levels in mice (*p < *0.05). Compared with Control (M+), M2 and M3 fermented milk doses after the third week reduced TG by 78.9% and 56%, respectively. Like what Xie et al. [27] found, giving mice a high-cholesterol diet plus *L. plantarum* 9-41-A and *L. fermentum* M1-16 may lower their TGs. In the same way, Zhong et al. [35] showed that the *Lactobacillus casei* could lower TG in mice that consumed a large amount of cholesterol. Furthermore, *L. plantarum* reduces TG in a high-cholesterol-fed mouse diet [32,33]. Furthermore, Jang et al. [34] also stated that administering *P. acidilactici *FS2 in mice can reduce TG in the blood. Lim et al. [17] revealed that high-cholesterol diet mice supplemented by *L. plantarum *TAR4 showed significantly decreased TG serum (45.31%). Yang et al. [3] exposed decreasing TG serum to the hamster for 8 weeks after supplementing *L. fermentum *ZJUIDS06.

**Table 1.** Effect of fermented milk *P. acidilactici* BK01 addition to bodyweight.

Weeks	Group			
	M+	M1	M2	M3
1	196.50 ± 13.48	194.00 ± 8.45	215.00 ± 16.27	204.50 ± 12.40
2	201.75 ± 15.8	198.75 ± 9.5	220.00 ± 15.25	219.00 ± 9.83
3	196.00 ± 14.63^a^	189.00 ± 7.75	212.00 ± 9.93	232.25 ± 55.85

a No significat difference (*p *> 0.05).

**Table 2.** The effect of fermented milk *P. acidilactici* BK01 addition to total serum cholesterol.

Weeks	Group			
	M+	M1	M2	M3
1	56.00 ± 4.55^a^	46.00 ± 7.39^a^	48.00 ± 11.2^a^	41.25 ± 11^ab^
2	81.25 ± 14.03^ab^	74.50 ± 2.08^abc^	69.25 ± 13.07^abc^	77.25 ± 20.71^abc^
3	70.00 ± 6.68^abc^	63.50 ± 13.23^abcd^	61.25 ± 13.23^abcd^	48.50 ± 6.45^abcd^

^a,b,c.d^ Significant differences (*p *< 0.05) are represented by different letters within the same column.

**Table 3.** Effect of fermented milk *P. acidilactici* BK01 addition to HDL cholesterol.

Weeks	Group			
	M+	M1	M2	M3
1	35 ± 4.16^a^	35.75 ± 4.92^ab^	32 ± 2.45^ab^	29 ± 4.97^ab^
2	46.5 ± 7.55^ab^	59.5 ± 12.12^ab^	31.75 ± 12.09^ab^	40.5 ± 10.34^ab^
3	44 ± 11.28^ab^	45.75 ± 10.72^b^	39 ± 5.23^b^	34 ± 4.32^b^

^a,b.^Significant differences (*p *< 0.05) are represented by different letters within the same column.

**Table 4.** The effect of fermented milk *P. acidilactici* BK01 addition to TGs.

Weeks	Group			
	M+	M1	M2	M3
1	125.5 ± 8.74^a^	144.25 ± 17.11^a^	104.25 ± 6.13^ab^	146.75 ± 22.91^abc^
2	156 ± 14.99^abc^	171.25 ± 41.88^abcd^	165.75 ± 43.69^abcd^	117.75 ± 30.85^abcd^
3	96.75 ± 15.78^bcde^	85.75 ± 13.94^cde^	72.5 ± 20.47^de^	56.75 ± 11^de^

^a,b.c.d.e.f.^Significant differences (*p *< 0.05) are represented by different letters within the same column.

According to Yamaoka et al. [36], decreasing TGs is a possible mechanism. It falls into bile acid-binding that returns to the liver through the enterohepatic cycle and promotes cholesterol to bile acid conversion. Reducing TGs inhibits the synthesis of fatty acids in the liver by enhancing the creation of short-chain fatty acids. According to Cavallini et al. [37], the fatty acid-forming enzyme acetyl CoA carboxylase can be inhibited by LAB. This enzyme will decrease the synthesis of fatty acids and automatically drop TG levels. Harisa et al. [38] propose that probiotics’ hypotriglyceridemic impact is related to lipase activity, decreased intestine lipid absorption, enhanced lipid catabolism, and/or antioxidant activity. Lipoprotein lipase regulates TG metabolism and plasma levels. Furthermore, the decrease in TGs is also associated with the metabolic pathways of the digestive microbiota in producing choline [39]. When cholin is converted into lecithin, it excretes low-density lipoprotein and prevents it from accumulating in the liver [40], so it can reduce TGs.

Several factors have a significant impact on the effect that probiotic supplements have on propyl lipids, including differences in the duration of probiotic administration, the strain of probiotics, dosage, and the characteristics of the research subjects. It can be concluded that probiotics can increase lipid metabolism by lowering total TGs. However, further research is necessary to determine the efficacy of probiotics for lowering propyl cholesterol and other lipids.

### LAB in the small intestine

**Figure 1.**
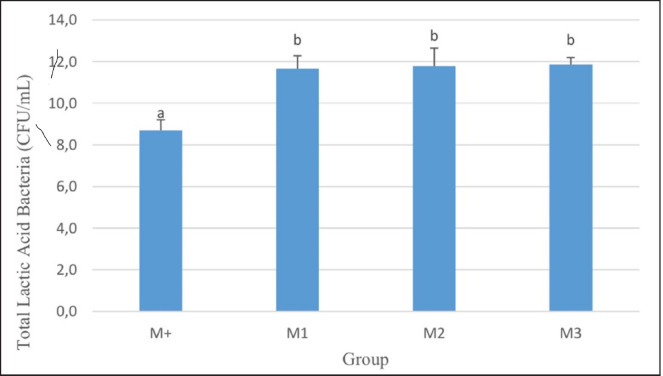
*Pediococcus acidilactici* BK01’s effect on small intestine LAB.

*Pediococcus acidilactici* BK01 fermented milk significantly (*p* < 0.05) increased total LAB in the small intestine after week 3 compared to the control (M+) (Fig. 1). Giving 1.05 ml (M3) resulted in a 36.78% increase in the total LAB compared to the control group. It showed that LAB could survive and multiply in the intestine. According to Saarela et al. [41], one of the factors for selecting potential probiotic strains is the bacteria’s capacity to cling to epithelial cell surfaces. Probiotic strain adhesion on mucosal surfaces is one of the essential probiotic features since it is frequently recognized as a requirement for colonizing the human gastrointestinal tract. Melia et al. [24] determined that *P. acidilactici* BK01 was a potential probiotic due to its resistance to acidic conditions, bile salts, and ability to suppress pathogenic bacteria. Like the study of Salaj et al. [42], *L. plantarum* LS/07 and *Biocenol* LP96 increased LAB numbers in the caecum relative to the control. Zafar et al. [21] showed recolonization of gut microbiota in the intestine or colon. Re-colonization of *L. fermentum* FM6 and *L. rhamnosus* Y59 for 2.55 × 10^7^ CFU/gm had a maximum gain on the colon.

**Figure 2.**
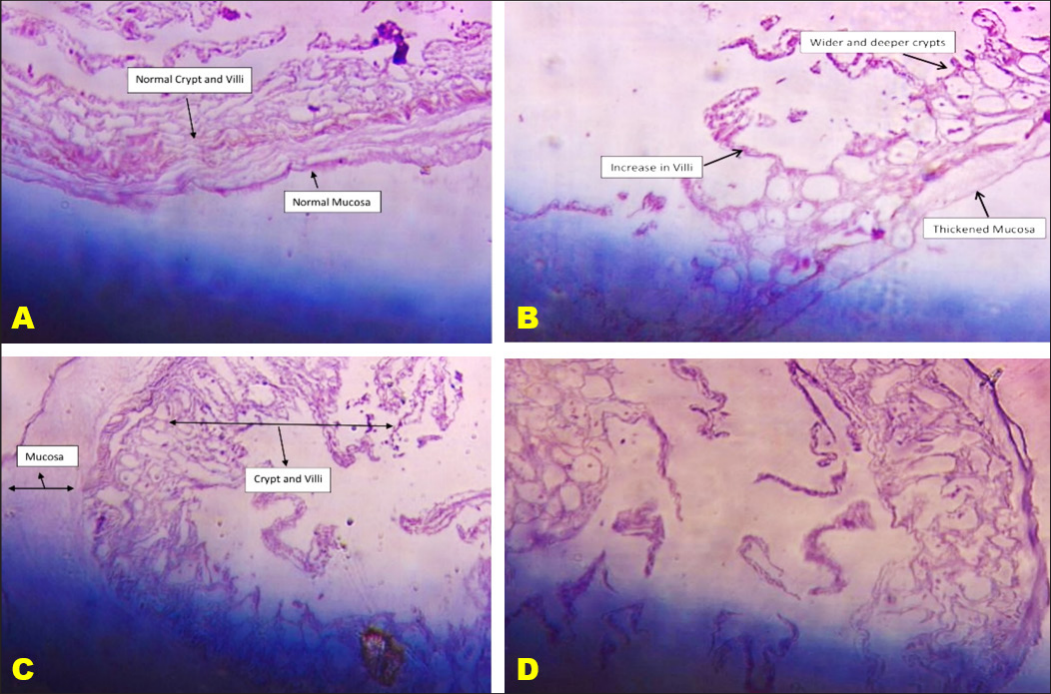
(A) Group M+, (B) Group M1, (C) Group M2, and (D) Group M3. Intestines of control mice had normal intestinal villi and normal intestinal mucosa. There was no damage to the intestinal villi. At the M1 doses, the thickness of the intestinal mucosa treated with probiotics had thickened, and the villi height and crypt depth were also seen.

### Small intestine histology

Figure 2 showed that the intestines of control (M+) mice had normal intestinal villi and normal intestinal mucosa. There was no damage to the intestinal villi. Furthermore, at the M1 doses, the thickness of the intestinal mucosa treated with probiotics had thickened, and the villi height and crypt depth were also seen. Following research conducted by Wresdiyati et al. [43], *L. plantarum* had a better effect on the thickness of the ileal mucosa. In addition, Smith et al. [44] reported that the administration of *L. fermentum* BR11 in mice could increase intestinal mucus thickness.

Furthermore, probiotics could induce short-chain fatty acids. The increase in short-chain fatty acids stimulates the proliferation of epithelial cells. The longer the treatment with probiotics, the thicker the intestinal mucosa and the more intestinal villi there are. Probiotics contribute to the wellness and protection of the host’s intestinal mucosa in several ways. For example, as seen in the M2 and M3 treatments, they attach epithelial cells to the surface of the host’s gastrointestinal system. Microbial adhesion to epithelial cells is regarded as one of the most implemented solutions for probiotic strain selection criteria. Palaniyandi et al. [22] explained that Caco-2 cells were used as a model to explain the adherence of putative probiotics to intestinal epithelial cells. *L. fermentum *MJM60397 exhibited greater adherence to the Caco-2 cell monolayer than LGG in this research. *Lactobacillus* strains bind to specific cell surface receptors, explaining their high adherence.

However, in this study, a fermented milk *P. cidilactici *BK01 supplement for 3 weeks only reduced total cholesterol and TGs and increased LAB total in the intestine mucosa. Yet affected by HDL. In addition, it is necessary to explore further the possibility of increasing the dose or extending the duration of application (Fig. 2). Small intestine staining results with 100× dilatation. (M+). normal villous and crypts, and normal mucosa. (M1) crypts were dilated, intestinal mucosa was thickened, and intestinal villi length was increased. (M1). Thickening of the mucosa and increased intestinal villi length (M3). Intestinal villi were well-developed and filled the intestinal lumen.

## Conclusion

To sum up, this study revealed *P. acidilactici* BK01 fermented milk had no effect on body weight or HDL. However, it has a beneficial effect on total serum cholesterol and TG levels. Additionally, treatment of fermented milk *P. acidilactici* BK01 has been shown to increase the total amount of LAB in the intestine, as indicated by changes in the intestinal villi. 

## List of abbreviations

LAB, Lactic acid bacteria; MRS, de Man Rogosa Sharpe; HDL, High-Density Lipoprotein; TG, Triglycerides.

## References

[1] Dicks LMT, Botes M. (2010). Probiotic lactic acid bacteria in the gastro-intestinal tract: health benefits, safety and mode of action. Benef Microbes..

[2] Tsai YT, Cheng PC, Pan TM. (2014). Anti-obesity effects of gut microbiota are associated with lactic acid bacteria. Appl Microbiol Biotechnol.

[3] Yang D, Lyu W, Hu Z, Gao J, Zheng Z, Wang W (2021). Probiotic effects of *Lactobacillus fermentum* ZJUIDS06 and *Lactobacillus plantarum* ZY08 on hypercholesteremic golden hamsters. Front Nutr.

[4] Nagpal R, Kumar A, Kumar M, Behare P V, Jain S, Yadav H. (2012). Probiotics, their health benefits and applications for developing healthier foods: a review. FEMS Microbiol Lett.

[5] Roza E, Aritonang SN, Yellita Y, Susanty H, Rizqan, Pratama YE. (2021). Potential of dadiah kapau from Agam district, Indonesia as a source of probiotics for health. Biodiversitas.

[6] Wirawati CU, Sudarwanto MB, Lukman DW, Wientarsih I, Srihanto EA. (2019). Diversity of lactic acid bacteria in dadih produced by either back-slopping or spontaneous fermentation from two different regions of West Sumatra, Indonesia. Vet World.

[7] Pato U, Yusuf Y, Nainggolan YP. (2019). Effect of *Lactobacillus casei subsp. casei* R-68 isolated from dadih on the procarcinogenic enzyme activity and fecal microflora count of rats challenged with pathogenic bacteria. Int J Adv Sci Eng Informat Technol.

[8] Nuraida L. (2015). A review: health promoting lactic acid bacteria in traditional Indonesian fermented foods. Food Sci Human Well.

[9] Alzahra H, Susmiati, Melia S. (2022). Evaluation of *Lactiplantibacillus pentosus* probiotic fermented buffalo milk with citrus juice. Adv Vet Anim Sci.

[10] Wikandari PR, Suparmo S, Marsono Y, Rahayu ES. (2012). Characterization of proteolytic lactic acid bacteria in Bekasam. J Nat Indonesia.

[11] Melia S, Purwati E, Kurnia YF, Pratama DR. (2019). Antimicrobial potential of *Pediococcus acidilactici* from Bekasam, fermentation of sepat rawa fish (*Tricopodus trichopterus*) from Banyuasin, South Sumatra, Indonesia. Biodiversitas.

[12] Juliyarsi I, Hartini P, Yuherman DA, Arief PH, Aritonang SN, Hellyward J (2018). Characterization of lactic acid bacteria and determination of antimicrobial activity in Tempoyak from Padang Pariaman District, West Sumatra, Indonesia. Pak J Nutr.

[13] Syukur S, Safrizayanti, Zulaiha S, Supriwardi E, Fahmi A, Nurfadilah KK (2022). Potential antioxidant activity of *Lactobacillus fermentum *KF7 from virgin coconut oil products in Padang, West Sumatra, Indonesia. Biodiversitas.

[14] Moon YJ, Baik SH, Cha YS. (2014). Lipid-lowering effects of *Pediococcus acidilactici* M76 isolated from Korean traditional makgeolli in high fat diet-induced obese mice. Nutrients.

[15] Le B, Yang SH. (2019). Identification of a novel potential probiotic *Lactobacillus plantarum* FB003 isolated from salted-fermented shrimp and its effect on cholesterol absorption by regulation of NPC1L1 and PPARα. Probiot Antimicrob Prot.

[16] Yamasaki M, Minesaki M, Iwakiri A, Miyamoto Y, Ogawa K, Nishiyama K (2020). *Lactobacillus plantarum* 06CC2 reduces hepatic cholesterol levels and modulates bile acid deconjugation in Balb/c mice fed a high-cholesterol diet. Food Sci Nutr.

[17] Lim PS, Loke CF, Ho YW, Tan HY. (2020). Cholesterol homeostasis associated with probiotic supplementation *in vivo*. J Appl Microbiol.

[18] Zhao L, Shen Y, Wang Y, Wang L, Zhang L, Zhao Z (2022). *Lactobacillus plantarum* S9 alleviates lipid profile, insulin resistance, and inflammation in high-fat diet-induced metabolic syndrome rats. Sci Rep.

[19] Yadav R, Khan SH, Mada SB, Meena S, Kapila R, Kapila S. (2019). Consumption of probiotic *Lactobacillus fermentum* MTCC: 5898-fermented milk attenuates dyslipidemia, oxidative stress, and inflammation in male rats fed on cholesterol-enriched diet. Probiot Antimicrob Prot.

[20] Wang M, Zhang B, Hu J, Nie S, Xiong T, Xie M. (2020). Intervention of five strains of *Lactobacillus *on obesity in mice induced by high-fat diet. J Funct Foods.

[21] Zafar H, Ul AN, Alshammari A, Alghamdi S, Raja H, Amjad Siddique A (2021). *Lacticaseibacillus rhamnosus *FM9 and *Limosilactobacillus fermentum *Y57 are as effective as statins at improving blood Wistar rats. Nutrients.

[22] Palaniyandi SA, Damodharan K, Suh JW, Yang SH. (2020). Probiotic characterization of cholesterol-lowering *Lactobacillus fermentum* MJM60397. Probiot Antimicrob Prot.

[23] Daliri EBM, Kim Y, Do Y, Chelliah R, Oh DH. (2022). *In vitro* and *in vivo* cholesterol reducing ability and safety of probiotic candidates isolated from Korean fermented Soya beans. Probiot Antimicrob Prot.

[24] Melia S, Juliyarsi I, Kurnia YF, Pratama YE, Pratama DR. (2014). The quality of fermented goat milk produced by *Pediococcus acidilactici* BK01 on refrigerator temperature. Biodiversitas.

[25] Damodharan K, Lee YS, Palaniyandi SA, Yang SH, Suh JW. (2015). Preliminary probiotic and technological characterization of *Pediococcus pentosaceus* strain KID7 and *in vivo* assessment of its cholesterol-lowering activity. Front Microbiol.

[26] Ulrike E, Christoph Loddenkemper KD, Simone Spieckermann DH, Markus Heimesaat M, Zeitz1 M, Britta Siegmund AAK. (2014). A guide to histomorphological evaluation of intestinal inflammation in mouse models. Int J Clin Exp Pathol.

[27] Xie N, Cui Y, Yin YN, Zhao X, Yang JW, Wang ZG (2011). Effects of two *Lactobacillus* strains on lipid metabolism and intestinal microflora in rats fed a high-cholesterol diet. BMC Complement Altern Med.

[28] Chen Y, Yang N, Lin Y, Ho S, Li K, Lin J (2018). A combination of *Lactobacillus mali *APS1 and dieting improved the efficacy of obesity treatment via manipulating gut microbiome in mice. Sci Rep.

[29] Molinero N, Ruiz L, Sánchez B, Margolles A, Delgado S. (2019). Intestinal bacteria interplay with bile and cholesterol metabolism: implications on host physiology. Front Physiol.

[30] Baccouri O, Boukerb AM, Farhat LB, Zébré A, Zimmermann K, Domann E (2019). Probiotic potential and safety evaluation of *Enterococcus faecalis* OB14 and OB15, isolated from traditional Tunisian Testouri Cheese and Rigouta, using physiological and genomic analysis. Front Microbiol.

[31] Shehata MG, El-Sahn MA, El Sohaimy SA, Youssef MM. (2019). *In vitro* assessment of hypocholesterolemic activity of *Lactococcus lactis* subsp. lactis. Bull Nat Res Centre.

[32] Ding W, Shi C, Chen M, Zhou J, Long R, Guo X. (2017). Screening for lactic acid bacteria in traditional fermented Tibetan yak milk and evaluating their probiotic and cholesterol-lowering potentials in rats fed a high-cholesterol diet. J Funct Foods.

[33] Huang Y, Wang X, Wang J, Wu F, Sui Y, Yang L (2013). *Lactobacillus plantarum* strains as potential probiotic cultures with cholesterol-lowering activity. J Dairy Sci.

[34] Jang WJ, Kim CE, Jeon MH, Lee SJ, Lee JM, Lee EW (2021). Characterization of *Pediococcus acidilactici* FS2 isolated from Korean traditional fermented seafood and its blood cholesterol reduction effect in mice. J Funct Foods.

[35] Zhong Z, Zhang W, Du R, Meng H, Zhang H. (2012). *Lactobacillus casei* Zhang stimulates lipid metabolism in hypercholesterolemic rats by affecting gene expression in the liver. Eur J Lipid Sci Technol.

[36] Yamaoka-Tojo M, Tojo T, Izumi T. (2008). Beyond cholesterol lowering: pleiotropic effects of bile acid binding resins against cardiovascular disease risk factors in patients with metabolic syndrome. Curr Vasc Pharmacol.

[37] Cavallini DC, Bedani R, Bomdespacho LQ, Vendramini RC, Rossi EA. (2009). Effects of probiotic bacteria, isoflavones and simvastatin on lipid profile and atherosclerosis in cholesterol-fed rabbits: a randomized double-blind study. Lipids Health Dis.

[38] Harisa GI, Taha EI, Khalil AF, Salem MM. (2009). Oral administration of *Lactobacillus acidophilus* restores nitric oxide level in diabetic rats. Austr J Basic Appl Sci.

[39] Stremmel W, Schmidt KV, Schuhmann V, Kratzer F, Garbade SF, Langhans CD (2017). Blood trimethylamine-N-oxide originates from microbiota mediated breakdown of phosphatidylcholine and absorption from small intestine. PLos One.

[40] Mehedint MG, Zeisel SH. (2013). Choline’s role in maintaining liver function: new evidence for epigenetic mechanisms. Curr Opin Clin Nutr Metab Care.

[41] Saarela M, Mogensen G, Fondén R, Mättö J, Mattila-Sandholm T. (2000). Probiotic bacteria: safety, functional and technological properties. J Biotechnol.

[42] Salaj R, Štofilová J, Šoltesová A, Hertelyová Z, Hijová E, Bertková I (2013). The effects of two *Lactobacillus plantarum* strains on rat lipid metabolism receiving a high fat diet. Sci World J.

[43] Wresdiyati T, Laila SR, Setiorini Y, Arief II, Astawan M, Anatomi D (2011). Probiotik indigenus meningkatkan profil kesehatan usus halus tikus yang diinfeksi enteropathogenic *E. coli* indigenous. Bandung Med J.

[44] Smith CL, Geier MS, Yazbeck R, Torres DM, Butler RN, Howarth GS. (2008). *Lactobacillus fermentum* BR11 and fructo-oligosaccharide partially reduce jejunal inflammation in a model of intestinal mucositis in rats. Nutr Cancer.

